# The internal structure of Eris inferred from its spin and orbit evolution

**DOI:** 10.1126/sciadv.adi9201

**Published:** 2023-11-15

**Authors:** Francis Nimmo, Michael E. Brown

**Affiliations:** ^1^ Department of Earth and Planetary Sciences, University of California Santa Cruz, Santa Cruz CA 95064, USA.; ^2^ Division of Geological and Planetary Sciences, California Institute of Technology, Pasadena CA 91125, USA.

## Abstract

The large Kuiper Belt object Eris is tidally locked to its small companion Dysnomia. Recently obtained bounds on the mass of Dysnomia demonstrate that Eris must be unexpectedly dissipative for it to have despun over the age of the solar system. Here, we show that Eris must have differentiated into an ice shell and rocky core to explain the dissipation. We further demonstrate that Eris’s ice shell must be convecting to be sufficiently dissipative, which distinguishes it from Pluto’s conductive shell. The difference is likely due to Eris’s apparent depletion in volatiles compared with Pluto, perhaps as the result of a more energetic impact.

## Teaser

The dwarf planet Eris melted early in its history and still hosts a warm, convecting ice shell.

## INTRODUCTION

Much remains mysterious about Eris, the most massive dwarf planet known. Unlike its cousin Pluto, which was explored by the *New Horizons* spacecraft and revealed a dynamic and variegated world (*1*), only basic characteristics of Eris, such as its mass, mean radius, and surface composition (*2*–*4*), are available. In particular, its internal structure, for instance, whether it consists of a homogeneous rock-ice mixture or not, is unknown. Here, we use recent observations of its spin and orbital characteristics to constrain the internal structure of Eris. We find that in some respects, it resembles Pluto, but in other respects it is quite different, illustrating the extent of diversity among even nominally similar Kuiper Belt objects (KBOs).

The surface of Eris is unusually bright (*5*–*6*) and is dominated by methane and nitrogen (*4*, *7*). These effects likely arise because condensation of a preexisting atmosphere forms a surface frost; as a result, the composition of the surface beneath the frost layer is unknown. It could be an undifferentiated rock-ice carapace (*8*) or be primarily water ice, as at Pluto.

The internal structures of large KBOs remain largely unconstrained. Inferred densities (which are quite variable) provide information on the rock:ice ratio (*9*), while the rapid increase in the depth of water ice absorption features on KBOs larger than about ~750 km has been suggested to be due to differentiation and an early interior ocean for the larger KBOs, with later resurfacing with water ice as the oceans froze (*10*). In the case of Haumea, its shape has been interpreted as indicative of a differentiated body (*11*). Pluto is also interpreted to have differentiated into an icy shell and a rocky core, and there is circumstantial evidence for a subsurface ocean (*12*). The differentiation state of these bodies provides clues as to how, and how rapidly, they accreted (*13*), while the possible presence of subsurface oceans is important for understanding the habitability of the outer reaches of the solar system.

Two recent observations provide clues to Eris’s internal structure (see Table 1). The first is that the Eris-Dysnomia system, like Pluto-Charon, is doubly synchronous, indicating that Eris must be quite dissipative to have spun down over 4.5 billion years (Ga) (*14*, *15*). These studies were only able to provide limited quantitative analyses because the mass of Dysnomia, which provides the torque to spin Eris down, was not well determined. The second observation used Atacama Large Millimeter/Submillimeter Array astrometry to provide a firm upper bound on the mass of Dysnomia (mass ratio 0.0084 at 1-σ and 0.015 at 3-σ) (*16*). With this upper bound in hand, a lower bound can be placed on how dissipative Eris must be. This lower bound turns out to be unexpectedly informative and strongly suggests that Eris, like Pluto, is differentiated but, unlike Pluto, likely hosts a convecting ice shell.

**Table 1.** Present-day parameter values.

Parameter	Value	Reference
System mass (kg)	1.6466 × 10^22^	(*2*)
Dysnomia mass (kg)	<1.4 × 10^20^	(*16*)
Semi-major axis (km)	37,273	(*2*)
Spin/orbit period (hour)	378.862	(*2*)
Eris radius (km)	1163 ± 6	(*3*)
Dysnomia radius (km)	350 ± 58	(*46*)
Eris density (kg m^−3^)	2500 ± 39	(*3*)
Dysnomia density (kg m^−3^)	700 ± 500	(*16*)

The rest of this paper is organized as follows. We first review the arguments that enable us to quantify dissipation in Eris, as measured by the parameter *Q*/*k*_2_, and provide supporting orbital evolution calculations. We then use the *Q*/*k*_2_ value derived to make inferences about Eris’s internal state. Last, we summarize our findings and provide suggestions for future work.

## RESULTS

### Orbital evolution

The origin of the Eris-Dysnomia system is uncertain. However, the small secondary:primary mass ratio and the differing densities of the two bodies suggest a giant impact origin (*15*, *16*). As with the Pluto-Charon system (*17*), the two bodies were likely much closer together initially, but tides raised on the primary by the secondary spun the primary down and transferred angular momentum to the secondary, increasing its semi-major axis. This situation was analyzed by both (*14*) and (*15*); with the updated constraint on Dysnomia’s mass, we will rederive some of their results below, but our efforts are focused on the implications of the *Q*/*k*_2_ bounds obtained.

Given a mass for Dysnomia, the present-day angular momentum budget of the system is known. As long as no external torques are acting, the spin period of Eris as a function of the semi-major axis of Dysnomia can then be derived (assuming Dysnomia’s spin is synchronous). The rate at which the spin/orbital evolution happens depends on the *Q*/*k*_2_ of Eris, where *k*_2_ is the tidal Love number, which describes the size of the tidal response, and *Q* is the quality factor which describes how dissipative Eris is. A small *Q*/*k*_2_ indicates a dissipative object.

Equations describing the spin and orbital evolution of a binary system have been derived by various authors (*18*–*20*). Below we use the formulation of (*21*) (Materials and Methods), with the major simplifications being that we assume eccentricities are zero throughout [cf. (*22*)] and ignore the effect of any higher-order spin-orbit resonances (e.g., a 3:2 resonance). We initially assume that *Q* is constant but relax this assumption below. The nominal values used are given in Table 1 and we use a baseline mass ratio of 0.0084 (*16*). We assume an initial separation of 7 *R*_p_ (corresponding to an Eris rotation period of 31 hours, assuming angular momentum is conserved), where *R*_p_ is the primary radius, as representative of the immediate postimpact situation; the results below are insensitive to this parameter unless very wide initial separations are assumed. The results are also insensitive to the value of *Q* assumed for Dysnomia.

Numerical integration of the (*21*) equations shows that Eris just becomes synchronous at the present day (i.e., after 4.5 Ga) if *Q*/*k*_2_ is 3200. An earlier synchronization is possible but would require a lower (more dissipative) *Q*/*k*_2._

Bernstein *et al.* (*15*) derives an approximation for the synchronization time scale, which can be written as

$$t_{\mathrm{sync}}\approx 1500\;\mathrm{Ma}\left(\frac{0.0084}{q}\right)\left(\frac{Q/k_{2}}{1000}\right)$$ (1)

where *q* is the mass ratio. For a synchronization time scale of 4.5 Ga, this yields a *Q*/*k*_2_ of 3000, essentially identical to the result derived here from numerical integration. Szakats *et al.* (*14*) mentioned the possibility that Eris might have an “unconventionally low” *Q*, but preferred a solution in which Dysnomia was more massive than the determination of (*16*) now allows.

### Frequency-dependent *Q*

An advantage of the (*21*) approach is that it allows more complicated evolution scenarios to be evaluated. In particular, for viscoelastic materials such as ice, one would expect *Q* to vary approximately linearly with the forcing frequency (Ω − *n*), where Ω is the spin frequency of Eris and *n* is the mean motion (see below). In this case, Eris will have become progressively more dissipative as it approached synchronous rotation. To obtain the same 4.5 Ga synchronization time scale as before but with *Q* varying with the forcing frequency, we need to set the initial *Q*/*k*_2_ to be 6300, when the forcing period is 165 hours.

**Fig. 1.**
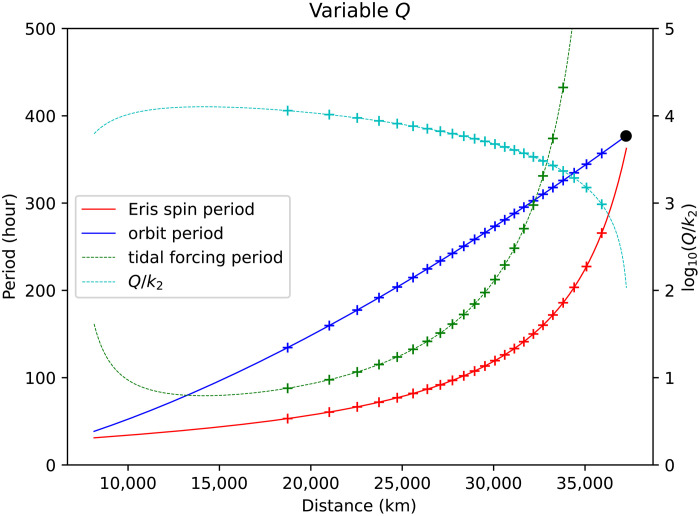
Evolution of Dysnomia’s orbital period and Eris’s spin period. We use the methodology of (*21*) and assume that Eris’s *Q* varies linearly with forcing frequency. The initial separation is 7*R*_p_ and Dysnomia is assumed synchronous throughout, with a mass ratio of 0.084. Crosses are at intervals of 200 Ma. The primary *k*_2_ is 0.12 and the *Q* is 760 for a forcing period of 165 hours. Other parameter values are given in Table 1; the black dot indicates the present-day system configuration.

Figure 1 plots the resulting evolution of the spin and orbit periods for the case that Dysnomia is synchronous throughout and *Q* for Eris varies linearly with forcing frequency. The spin and orbit curves (solid lines) are identical to the corresponding curves in (*15*), but the evolution rate is different. In this plot, synchronization is just attained after 4.5 Ga. The dashed lines depict the tidal forcing period and log(*Q*/*k*_2_) for Eris, showing that Eris becomes more dissipative as synchronous rotation is approached. As a result of the time-varying *Q*, the rate of outward evolution is nonmonotonic; the time-averaged *Q*/*k*_2_ is 5500, which is less than a factor of 2 different from the constant-*Q* value derived above. The advantage of the variable-*Q* model is that it is easier to interpret in terms of internal structure models, as discussed below.

Because we have only an upper bound on Dysnomia’s mass, Eris could be more dissipative than our baseline calculations indicate. For instance, if we make Dysnomia’s density 500 kg m^−3^ and start at 9 *R*_p_, then the time-averaged *Q*/*k*_2_ for Eris is 3500. Thus, our estimates of Eris’s dissipation are conservative.

### Internal structure

We assume a bulk density for Eris of 2500 kg m^−3^ (*3*), while that of Dysnomia is 700 ± 500 kg m^−3^ (*16*). The former value implies that Eris is composed primarily of rock. If the rock component had a density of 2500 kg m^−3^, comparable to that inferred for Enceladus’s core (*23*), the ice fraction would be ≈0%. At the other extreme, an entirely rocky/metallic core of density 3500 kg m^−3^, comparable to the bulk density of Io, would imply an ice mass fraction of 15%.

If Eris were a homogeneous mix of rock and ice, its material properties would therefore be dominated by the rock fraction. Because of its size, we will assume that Eris is not a rubble pile [which would imply a rather different tidal response (*24*)] but is monolithic. For a homogeneous body, we can calculate *k*_2_ directly

$$k_{2}\approx \frac{3\rho gR}{19\mu }$$ (2)

where *g* is the surface gravity, ρ the bulk density, *R* the radius of Eris, and μ the shear modulus of rock. From Table 1, we obtain *g* = 0.81 ms^−2^ and taking μ = 30 GPa, we find *k*_2_ ≈ 0.01. This in turn would require a *Q* of <30 or <60 to match the *Q*/*k*_2_ constraint at 1- or 3-σ in the case that *Q* is assumed constant.

Since *Q* is strongly temperature dependent, consideration of the temperature structure of a homogeneous Eris is warranted. For a conductive, steady-state, uniformly heated sphere, the central temperature is

$$T_{\mathrm{cen}}=T_{s}+\frac{\rho HR^{2}}{6k}$$ (3)

where *H* is the heat production rate and *k* is the thermal conductivity. The present-day chondritic heating rate is about 4.5 × 10^−12^ W kg^−1^ (*25*) so the present-day central temperature of Eris should be about 875 K, taking *k* = 3 Wm^−1^ K^−1^ and the surface temperature *T*_s_ = 30 K. This value suggests that ice melting and differentiation are viable (see below), in particular in the deep past when heat production was higher. However, the central temperature is also about 500 K below the melting temperature of rock, which would therefore have a viscosity of order 10^28^ Pa s. Such high viscosities imply negligible dissipation.

Our upper bound on *Q* may be compared with Mars and the Moon, which have *Q* values of ≈80 and ≈35, respectively (*26*, *27*). The Moon is dissipative because of its warm lower mantle and liquid metallic core (*26*). Because of its smaller size, Eris is expected to be colder and less dissipative than the Moon (see above). We conclude that an undifferentiated, rock-dominated Eris is not compatible with a constant *Q* of <60.

If Eris is differentiated, the situation is quite different. Ice is less rigid and has a lower viscosity than rock; it is therefore much more susceptible to tidal dissipation. A differentiated Eris would have an ice shell thickness *d* = 120 km, assuming an intermediate rock density of 3100 kg m^−3^. The rock core will not substantially deform, but the ice shell will, and can dissipate enough energy to explain Eris’s inferred *Q*/*k*_2_.

### Viscoelastic model

Rather than assuming that *Q* is constant, a better assumption is that dissipation is happening in a viscoelastic medium (*28*), in which case *Q*/*k*_2_ will be frequency dependent (as assumed in Fig. 1). We calculate the tidal response of a three-layer Eris assuming a Maxwellian viscoelastic rheology and the method of (*29*). The model consists of a 30-km-thick ice lithosphere overlying an isoviscous ice shell 90 km thick, atop a rigid silicate core.

**Fig. 2.**
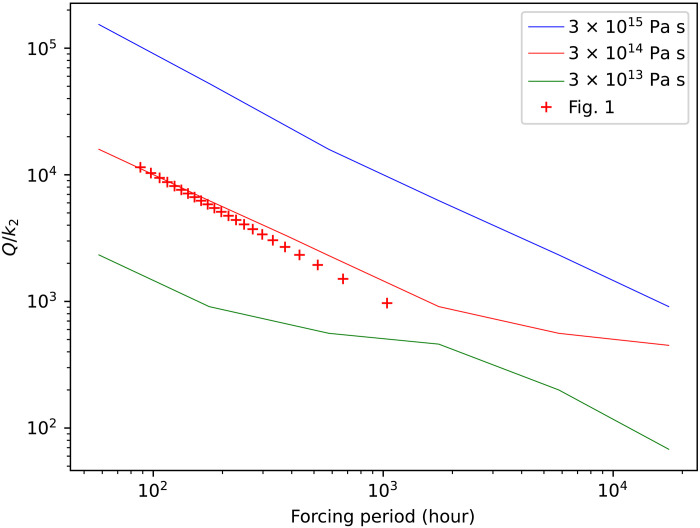
*Q/k*_2_ for a differentiated Eris as a function of forcing period and ice shell viscosity. We use the methodology of (*29*) and a Maxwellian rheology. The ice layer is 120 km thick with a density and rigidity of 950 kg m^−3^ and 3 GPa, respectively, while the silicate core is purely elastic with a rigidity of 30 GPa and a density of 3100 kg m^−3^. The ice lithosphere is purely elastic and 30 km thick; the remainder of the ice shell has a constant viscosity as noted in the figure label. Crosses denote the *Q*/*k*_2_ and forcing period values used in Fig. 1.

The solid lines in Fig. 2 show *Q*/*k*_2_ as a function of forcing period for this three-layer Eris for three different ice shell viscosities. Dissipation increases as viscosity decreases, and *Q*/*k*_2_ varies as approximately 1/period, as expected [the departures from linearity occur due to the transition from “fluid” to “solid” behavior (*28*)]. The crosses in Fig. 2 show the *Q*/*k*_2_ values used in Fig. 1. These crosses demonstrate that an ice shell viscosity in the range 1 to 3 × 10^14^ Pa s would provide the correct orbit evolution time scale.

Since the viscosity of ice near its melting point is expected to be in the range 10^13^ to 10^15^ Pa s (*30*), and recalling that our *Q*/*k*_2_ values are upper bounds, we conclude that a warm ice shell can explain the observations.

The natural way to explain a warm ice shell beneath a rigid lid is if the ice is convecting. In this situation, the bulk of the shell will be of a roughly constant viscosity, which must be sufficiently low that convection can occur. Quantitatively, the Rayleigh number (a dimensionless value describing the vigor of convection) is roughly 10^8^ for a viscosity of 3 × 10^14^ Pa s (Materials and Methods). The critical Rayleigh number for ice, which has a strongly temperature-dependent viscosity, is about 5 × 10^6^ for a Cartesian geometry (*31*). Thus, at least sluggish convection is expected, presumably driven by radiogenic heating (at a rate of about 5 mW m^−2^) from the silicate interior. Our orbital inference of a warm ice shell is therefore consistent with the physical requirements for convection to occur.

The presence of an assumed 30-km-thick lithosphere is consistent with the idea of ice shell convection (*32*) but is not required: Removing the lithosphere entirely would only change the inferred viscosity range by about 15%. However, an ice shell which was conductive rather than convective would be too cold and rigid to dissipate substantial energy. With a present-day heat flux of *F* = 5 mWm^−2^ and a mean ice thermal conductivity *k* = 4 Wm^−1^ K^−1^ (*33*), a conductive ice shell would have a basal temperature of $T_{b}\approx T_{s}+\left(\frac{Fd}{k}\right)\approx 180$ K, too low to permit important levels of dissipation.

This calculation also demonstrates that temperatures are likely too low to maintain a present-day subsurface ocean unless substantial quantities of antifreeze (such as NH_3_) were present. Heat fluxes will have been higher in the past (due to enhanced radiogenic heating and stored energy of accretion). However, in the case of Pluto, it has been shown that ice shell convection can remove heat efficiently enough that an ocean never forms (*31*), and the same may be true for Eris. If present beneath a convecting ice shell, an ocean would permit larger ice shell displacement, enhancing dissipation and making synchronization more rapid; but an ocean is not required by our results.

## DISCUSSION

To have synchronized over 4.5 Ga, Eris must be quite dissipative. An Eris differentiated into an ice shell and rocky core can explain its inferred dissipative properties, while a monolithic undifferentiated Eris cannot. The viscosity of the ice shell inferred requires the shell to be convecting, which is consistent with the calculated shell Rayleigh number. A subsurface ocean is not required by our results, and may never have formed if convection is vigorous enough.

### Simplifications

In Fig. 1, we made the simple assumption that *Q*^−1^ varies linearly with forcing period. Figure 2 shows that this assumption is a good approximation, at least for Maxwell viscoelasticity. Other rheological models provide arguably better descriptions of ice dissipative behavior (*34*), but these descriptions require more uncertain parameters to be specified. Furthermore, for the range of forcing periods of interest here, the dissipative characteristics overlap that of simple Maxwellian behavior (Materials and Methods) and are thus unlikely to modify our conclusions substantially.

Another simplification made in Fig. 1 is that it neglects any thermal evolution of Eris. At earlier times, Eris was probably hotter and potentially more dissipative. Although a full examination of the coupled thermal/orbital evolution problem is beyond the scope of this work, we carried out some simple calculations that crudely simulate a time-dependent *Q* arising from slow cooling as radiogenic heat production decays (Materials and Methods). In this case, the tendency of *Q*/*k*_2_ to decrease with time owing to the increasing forcing period (Fig. 1) is offset by the increase in *Q*/*k*_2_ due to the slow cooling of the ice shell. For a despinning time of 4.5 Ga, the model *Q*/*k*_2_ remains roughly constant at ≈4000 over the course of the simulation. Comparison with Fig. 2 shows that these results indicate an ice shell viscosity in the range of roughly 3 × 10^13^ to 3 × 10^15^ Pa s, not substantially different from our previous result. A more detailed model development would be desirable in future work.

We have also neglected eccentricity evolution. A nonzero present-day eccentricity for Dysnomia was reported by (*2*). If this value is correct, it is an indication that dissipation in Eris (which increases eccentricity) outweighs dissipation in Dysnomia (which decreases it) (*21*). Quantitatively, the *Q*/*k*_2_ of Dysnomia would have to be roughly 60 times larger (less dissipative) than that of Eris (Materials and Methods). Since Dysnomia’s small size leads to a smaller *k*_2_ (Eq. 2) and larger *Q* (it will be colder), this requirement is plausible.

Last, we have assumed a two-component (rock-ice) system for simplicity. If KBOs resemble comets, they could contain substantial fractions of carbonaceous material (*35*, *36*). These compounds would reduce the rate of radiogenic heat production and the thickness of the ice shell, making a subsurface ocean less likely; unfortunately, their dissipative properties are unknown.

### Implications and future work

Although both Eris and Pluto appear to be differentiated, Pluto is thought to have a conductive ice shell overlying an ocean while Eris’s ice shell is convective. Why the difference? To suppress convection on Pluto requires either cold ocean temperatures (*31*) or a layer of clathrates at the base of the shell (*37*), implying the presence of either NH_3_ or CH_4_, respectively. Thus, Eris may simply have a lower bulk abundance of these species than Pluto.

One possible explanation for this volatile depletion is that the Dysnomia-forming impact was more energetic than the impact that formed Charon (*38*). Eris’s higher density relative to Pluto (2.5 g/cc versus 1.85 g/cc) is also consistent with this idea: impact-driven removal of 15% of the icy mantle of a Pluto-like object would yield an Eris-like density. An alternative is that the high temperatures experienced during impacts (*19*) may have driven evaporative loss of a large fraction of Eris’s volatiles. Further modeling of the consequences of large KBO impacts (*17*, *39*) could be used to test these hypotheses.

Because convecting ice has a low viscosity, lateral variations in topography are hard to maintain (*40*). On Pluto, Sputnik Planitia is bright because it is a topographic low and thus a cold trap (*41*); we would not expect such a feature to survive on Eris and instead expect volatiles to be more uniformly distributed across the globe, which is consistent with Eris’s muted light curve compared to Pluto.

Another consequence of a convecting shell is that the shape of Eris should conform to that of an equipotential. Given Eris’s slow spin rate, the equipotential shape is basically a sphere; long-wavelength departures from sphericity (e.g., a large impact basin) would be hard to reconcile with our model of a convecting ice shell. Future measurements of the shape of Eris would thus be of great interest.

Last, while our results do not require a subsurface ocean, one possible consequence of such an ocean would be ocean pressurization and possible cryovolcanism as freezing progressed (*42*). Future detection of species that are short-lived (e.g., due to radiolysis) at the surface of Eris could be a possible indicator of such cryovolcanism.

## MATERIALS AND METHODS

### Orbital evolution model

We use (*21*) with the assumption that eccentricity is zero throughout and neglecting any complications arising from passage through a 3:2 spin:orbit resonance. We also assume that Dysnomia is synchronous throughout, as is appropriate given its much shorter despinning time scale. Under these circumstances we have

$$\left\langle \frac{d{\dot{\psi }}_{i}}{dt}\right\rangle =-\frac{3GM_{j}^{2}}{2C_{i}}\frac{k_{2i}}{Q_{i}}\frac{R_{i}^{5}}{a^{6}}[\mathrm{sgn}({\dot{\psi }}_{i}-n)]$$ (4)

$$a^{-1}\left\langle \frac{da}{dt}\right\rangle =3n\frac{k_{2i}}{Q_{i}}\frac{M_{j}}{M_{i}}{\left(\frac{R_{i}}{a}\right)}^{5}[\mathrm{sgn}({\dot{\psi }}_{i}-n)]$$ (5)

Here $\dot{\psi }$ is the rotation rate (Ω), *M* is mass, *R* is radius, *n* is the mean motion, *a* is the semi-major axis, and *C* is the polar moment or inertia. Subscript *i* refers to the primary (Eris) and *j* to the secondary (Dysnomia). We assume uniform density bodies such that the normalized *C* = 0.4. These equations are integrated using a simple first-order finite-difference scheme. For the frequency-dependent *Q* case, we specify *Q* at an initial angular forcing period and assume that *Q*^−1^ varies linearly with forcing period. The forcing period is calculated as 2π(Ω − *n*)^−1^.

### Convection and Rayleigh number

The Rayleigh number for a fluid with a strongly temperature-dependent viscosity is given by (*32*)

$$\mathrm{Ra}=\frac{\rho g\alpha \text{Δ}Td^{3}}{\kappa \eta_{b}}$$ (6)

Here ρ is density, α is thermal expansivity, ΔΤ is the temperature contrast across the fluid, *d* is the layer thickness, κ the thermal diffusivity and η_b_ the viscosity at the base of the fluid. For the case of Eris, approximate numbers are ρ = 950 kg m^−3^, α = 10^−4^ K^−1^, *d* = 120 km, *g* = 0.81 ms^−2^, and κ = 10^−6^ m^2^ s^−1^. If the base of the ice layer is close to the melting point, then Δ*T* ≈ 200 K. The resulting Rayleigh number is then ~10^8^ (3 × 10^14^ Pa s/η_b_).

### Maxwellian rheology

McCarthy and Cooper (*43*) show that an alternative description for the dissipative nature of ice is given by an Andrade rheology, where the local attenuation *Q*_loc_ is given by *J*_1_/*J_2_*, where we define

$$J_{1}=J_{u}+\beta \text{Γ}(1+\phi )\omega^{-\phi }\cos\left(\frac{\phi \pi }{2}\right)$$ (7)

$$J_{2}=\beta \text{Γ}(1+\phi )\omega^{-\phi }\sin\left(\frac{\phi \pi }{2}\right)+\frac{1}{\eta \omega }$$ (8)

Here *J*_u_ is the unrelaxed compliance (=1/shear modulus), β and ϕ are rheological parameters, Γ is the gamma function, ω is the forcing frequency and η is the steady-state viscosity. For ice, we have β = 4.5 × 10^−12^ Pa^−1^ s^−0.25^, ϕ = 0.25, and the shear modulus *E* is 3 GPa.

**Fig. 3.**
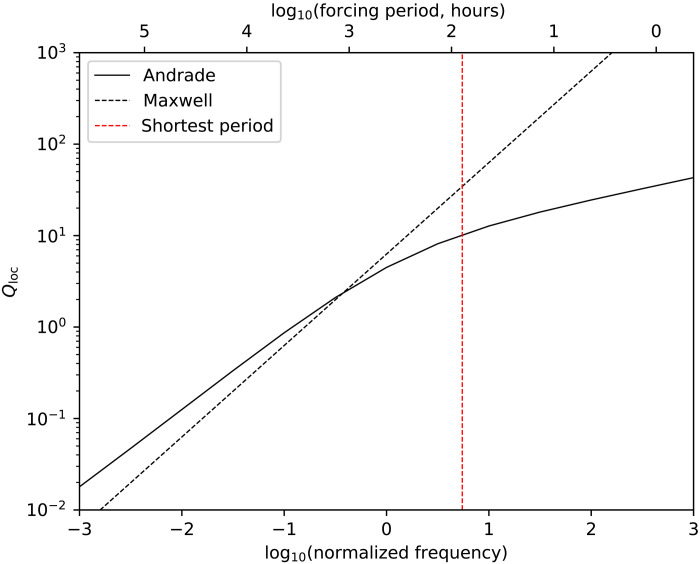
Local attenuation *Q*_loc_ as a function of frequency for Maxwell and Andrade rheologies. The forcing frequency is normalized to that of the Maxwell frequency (2π*E*/η) and calculations are carried out using the values given in the text and a steady-state viscosity of 1.4 × 10^16^ Pa s, based on (*43*). All of the forcing periods in our model are to the left of the red line.

Figure 3 below shows the local attenuation factor *Q*_loc_ as a function of forcing period for Maxwell and Andrade rheologies, where the shortest period in our model (Fig. 1) is shown with the dotted red line. At periods longer than this, the difference between the Andrade and Maxwell *Q*_loc_ is always less than an order of magnitude. Also note that the local *Q*_loc_ calculated here cannot be related in a simple manner to the bulk *Q* of the body shown in Figs. 1 and 2, because the local *Q*_loc_ does not take gravity or rigidity into account.

### Eccentricity of Dysnomia

The equations in (*21*) show that the eccentricity of the secondary grows or decays depending on the sign of the following quantity

$$\frac{57}{8}-\frac{21}{2}\frac{k_{2j}/Q_{j}}{k_{2i}/Q_{i}}\frac{R_{i}\rho_{i}^{2}}{R_{j}\rho_{j}^{2}}$$ (9)

The ratio (*R_i_*ρ*_i_*^2^/*R_j_*ρ*_j_*^2^) is uncertain but is roughly 40 (Table 1). For the quantity to be positive (increasing eccentricity), the *k*_2_/*Q* for Dysnomia then has to be about 60 times smaller than that for Eris.

### Time-dependent *Q*

We wish to develop a simple expression for how the viscosity and *Q* of the ice shell will react to the decreasing radiogenic heat production. If the shell is convecting, then the convective heat flux *F* goes as η^−1/3^, where η is the basal viscosity of the ice shell (*44*). We will assume that this basal viscosity adjusts itself so that *F* matches the rate at which heat is escaping from the silicate core, i.e., the system is in quasi-steady state. This heat will be dominated by radiogenic production, which has an effective decay constant of roughly 0.43 Ga^−1^ (*45*). With these assumptions, the viscosity of the ice shell will then increase exponentially with a time constant of 3 × 0.43 = 1.29 Ga^−1^. Away from the Maxwell peak, *Q* will increase linearly with viscosity (Fig. 2) and thus exhibit the same exponential increase with time. We can add this time-dependent factor for *Q* directly into the equations governing the spin and orbit evolution (see above).
